# Sequential Multilingualism and Cognitive Abilities: Preliminary Data on the Contribution of Language Proficiency and Use in Different Modalities

**DOI:** 10.3390/bs9090092

**Published:** 2019-08-26

**Authors:** Marlijne Boumeester, Marije C. Michel, Valantis Fyndanis

**Affiliations:** 1 Department of Languages, Literature and Communication, Utrecht University, 3512 JE Utrecht, The Netherlands; 2 Department of Applied Linguistics, Groningen University & Department of Linguistics and English Language, Lancaster University, Lancaster LA1 4YW, UK; 3 Department of Communication Sciences and Disorders, Long Island University, Brooklyn Campus, New York, NY 11968, USA; 4 MultiLing/Department of Linguistics and Scandinavian Studies, University of Oslo, 0315 Oslo, Norway

**Keywords:** multilingualism, cognitive abilities, inhibition, switching, disengagement of attention

## Abstract

This exploratory study focuses on sequential bi-/multilinguals (specifically, nonimmigrant young Dutch native speakers who learned at least one foreign language (FL) at or after the age of 5) and investigates the impact of proficiency-based and amount-of-use-based degrees of multilingualism in different modalities (i.e., speaking, listening, writing, reading) on inhibition, disengagement of attention, and switching. Fifty-four participants completed a comprehensive background questionnaire, a nonverbal fluid intelligence task, a Flanker task, and the Trail Making Test. Correlational and regression analyses considering multilingualism related variables and other variables that may contribute to the cognitive abilities under investigation (e.g., years of formal education, socioeconomic status, physical activity, playing video-games) revealed that only proficiency-based degrees of multilingualism impacted cognitive abilities. Particularly, mean FL writing proficiency affected inhibition (i.e., significant positive flanker effect) and L2 listening proficiency influenced disengagement of attention (i.e., significant negative sequential congruency effect). Our findings suggest that only those speakers who have reached a certain proficiency threshold in more than one FL show a cognitive advantage, which, in our sample, emerged in inhibition only. Furthermore, our study suggests that, regarding the impact of proficiency-based degrees of multilingualism on cognitive abilities, for our participants the writing and listening modalities mattered most.

## 1. Introduction

In our increasingly connected world, there is a growing number of people who are raised with more than one language or who learn a new language later in life. In fact, it has been estimated that more than half of the world’s population is multilingual to some extent [1].

A question that has been the topic of many studies is whether growing up as bi-/multilingual has an effect on cognitive abilities, such as executive functions. Executive functions (also referred to as executive or cognitive control) are defined as higher order, domain general, cognitive processes that regulate behavior and other cognitive processes such as attention and visual perception. According to the influential model formulated by Miyake et al. [2] (an updated version of this model is described in Miyake and Friedman [3]), there are three separable executive functions: (1) Switching, the ability to switch between two or more tasks or stimuli; (2) updating, the ability to monitor representations in working memory and replace no longer relevant representations with newer, more relevant ones; and (3) inhibition, the ability to deliberately suppress dominant or automatic responses, when necessary.

Many studies reported better performance of bi-/multilinguals than monolinguals on executive control tasks (recent reviews in [4,5,6]), such as the Flanker task, e.g., [7,8], the Stroop task, e.g., [9], and the Trail Making task, e.g., [10,11]. Although bi-/multilinguals throughout their whole lifespan have been found to outperform monolinguals on these tasks, the most consistent results have been found with children and older adults [5]. Studies with participants speaking a dialect in addition to their native language report similar results, e.g., [12,13].

The findings above suggest that the ability to speak in more than one language could lead to cognitive changes. This has traditionally been explained by the Inhibitory Control Model for Language Selection proposed by Green [14]. According to this model, bi-/multilinguals are constantly inhibiting activated lemmas from the non-target language during speech production in order to prevent intrusion from the non-target language into the target language. It is this continuous training of suppressing the non-target language that enhances domain general inhibitory control processes. Support for this theory came from studies showing that, in bi-/multilinguals, words from multiple languages are constantly active during language production, even in contexts where only one language is required, e.g., [15,16], review in [17]. Further empirical support for Green’s [14] model, and in particular for the idea that a domain-general system is recruited for language selection and control, was provided by neuroimaging studies reporting an overlap in brain networks involved in nonverbal task switching and language selection, e.g., [18,19,20]. Green’s [14] theory became the dominant explanation for bi-/multilingual effects on cognition and paved the way for many studies looking into these effects and exploring the implications of Green’s [14] Inhibitory Control Model for Language Selection (review in e.g., [5,21,22,23]. It is worth noting that, recently, Green and Abutalebi [24] updated Green’s [14] model proposing the Adaptive Control Hypothesis, which provides a more detailed description of the processes involved in bi-/multilingual language selection and the ramifications for cognition. In particular, Green and Abutalebi [24] distinguished eight control processes (goal maintenance, conflict monitoring, interference suppression, salient cue detection, selective response inhibition, task disengagement, task engagement, opportunistic planning) and argued that these processes are differentially recruited depending on the type of interactional context (single language, dual language, dense code-switching) for language use. According to the Adaptive Control Hypothesis, each of the three interactional contexts above poses differential demands on selection and control processes, as well as on the neural regions and circuits subserving these processes. As Bialystok [5] p. 251 notes, Green and Abutalebi’s [24] model “provides a promising way for understanding the essential role of the environment in shaping cognitive systems […] but final judgment on this model awaits further research”.

However, there are also several studies that did not find differences between monolinguals and bi-/multilinguals on executive function tasks, e.g., [25,26,27,28,29], see also a meta-analytic review by Lehtonen et al. [30]. These findings have led researchers to either propose that bi-/multilingualism does not lead to cognitive changes at all, or to question whether Green’s [14] model correctly explains how multilingualism could lead to cognitive changes. For example, studies finding bi-/multilinguals to be faster than monolinguals not only on incongruent trials, but also on congruent trials, where no inhibitory control is required, e.g., [7,31], led Costa et al. [7] to propose that bi-/multilingualism also has an effect on monitoring processes. Monitoring processes are used to determine whether mechanisms to resolve conflict are required during a trial, with better monitoring skills leading to faster responses on both congruent and incongruent trials. Monitoring abilities are enhanced in bi-/multilinguals because, unlike monolinguals, they constantly monitor which language is the most appropriate in each communicative setting, depending on the language knowledge of the conversation partner.

Furthermore, recent studies reported enhanced cognitive performance in preverbal infants growing up in a bi-/multilingual environment compared to infants growing up in a monolingual environment [32,33,34]. Based on these findings, Bialystok and colleagues [5,8,35] recently proposed changes to the prevailing theory on how multilingualism could affect cognitive functions. Since preverbal infants do not produce language yet, the bilingual advantage observed in these infants contradicts the idea that the enhanced executive functioning observed in verbally developed bi-/multilinguals results from the constant inhibition of the non-target language during speech. Bialystok [5], therefore, argued that multilingualism alters the way attention is directed to the environment. That is, because multilingual infants receive input in two or more languages that differ from each other in various aspects, they learn to focus their attention on the contrasts between the two systems. This leads bi-/multilinguals to attend more carefully to subtle environmental differences and ultimately improves their attentional processing [5]. Related to this, Grundy et al. [8] argued that multilingual input makes it more advantageous for the bi-/multilingual infant to be able to disengage attention from input once it has been processed in order to refocus attention to currently relevant input. To this end, Grundy et al. [8] tested the disengagement of attention ability in monolinguals and bilinguals by calculating the so-called sequential congruency effect (SCE). This measure captures how trial performance is affected by the congruency status of a preceding trial. That is, trials after a congruency switch (e.g., an incongruent trial after a congruent trial) typically take longer than trials with the same “(in)congruency status” as the previous trial. While Grundy et al. [8] found no differences between monolinguals and bilinguals on the traditional flanker effect, the bi-/multilinguals had lower SCEs than monolinguals, suggesting that they were less influenced by previous trial information and, therefore, better able to disengage their attention. The studies showing preverbal multilingual infants have enhanced cognitive abilities also suggest that not only oral production of languages other than L1, but also being exposed to or attending to languages other than L1 might matter. One could attempt to extend Green’s [14] model by assuming that inhibition of the non-target language(s) is not only dominantly involved in *speaking* but also in other modalities such as *listening*, *writing*, *and reading*. In fact, recent studies took into account different language modalities in order to compute participants’ degree of bi-/multilingualism [36,37].

Research into the relationship between bi-/multilingualism and cognitive abilities is further complicated by a couple of facts, possibly contributing to the contradictory results. First of all, many studies involve confounding factors, that is, factors that can also have an independent effect on executive functions, such as lifestyle (e.g., music training, playing videogames, and physical activity), education, socioeconomic status (SES), immigration status, and cultural differences, cf. [28,38,39,40,41]. Paap et al. [28] discuss that, in some studies, results showing a bilingual advantage could actually be explained by confounding variables that were not controlled for. For example, in several studies the group of bi-/multilinguals consisted of or included immigrants. Fuller-Thomson and Kuh ([40], p. 129) argue that there is a “healthy migrant effect”, which means that “healthier people are more likely to decide to migrate”. Given that the healthy migrant effect has been associated with increased cognitive control and slower rates of cognitive decline, e.g., [42], it is important to control for these confounding variables. Addressing Paap et al.’s [28] concerns, De Bruin, Bak and Della Sala’s [43] conducted a well-controlled study, in which groups of monolinguals and active and nonactive bilinguals were carefully matched on many potential confounding factors such as immigration status, lifestyle, SES, IQ and gender. In this study, there was no between-group difference in performance on executive control tasks.

Another aspect complicating the line of research on the relationship between bi-/multilingualism and cognitive abilities is the large variety of definitions of bi-/multilingualism in earlier work. For instance, participants have been classified as bi-/multilingual based on starting age of daily usage of more than one language [44], age of immersion in the second language (L2) environment [45], age of L2 fluency [46], balanced use of two languages [47], and a combination of age of acquisition and language proficiency [31], revealing the inconsistency and uncertainty of the plethora of criteria used in different studies. In fact, Luk and Bialystok [48] argue that bi-/multilingualism should not be seen as a categorical, static variable but as a composite of multiple dynamic and interrelated factors pertaining to language proficiency and use (see also [5,6]). Accordingly, bi-/multilingualism is not a single concept, as individuals can differ substantially from each other on all these multi-faceted factors that make up the concept of bi-/multilingualism. Consequently, it is problematic to create groups of bi-/multilinguals to compare them with monolinguals, since such an approach cannot capture the variability that will exist within these groups. To address these concerns, recent work has started to treat bi-/multilingualism no longer as a categorical homogeneous variable, but as a continuous variable, according to which participants are placed on a scale from less to more bi-/multilingual based on second/foreign language proficiency and amount of use. Under this approach, each participant is assigned an individual score representing their proficiency-based or amount-of-use-based degree of bi-/multilingualism [13,37,49,50].

### 1.1. Simultaneous vs. Sequential Bi-/Multilingualism and Executive Functioning

Most studies on the impact of bi-/multilingualism on cognitive abilities focused on bi-/multilinguals who learned two or more languages during infancy or early childhood (i.e., before age 5, as is a common definition in Second Language Acquisition (SLA); see [51]. This group will be referred to as *simultaneous bi-/multilinguals* (e.g., [52]). In recent years, there has been a growing interest in the question whether learning a new language at later ages could lead to cognitive advantages similar to those that have been found in simultaneous bi-/multilinguals.

One aspect that often distinguishes simultaneous bi-/multilinguals from sequential bi-/multilinguals is language competence: Typically, simultaneous bi-/multilinguals are assumed to reach native competence in the languages they are acquiring, whereas sequential bi-/multilinguals do not (e.g., [53]). Flege et al. [53] showed that the age of arrival in a new language environment was related with strength of foreign accents and knowledge of morphosyntactic rules. The difference in language competence is also reflected in self-rated proficiency scores, where simultaneous bi-/multilinguals score their target language proficiency higher than sequential bi-/multilinguals (e.g., [44,45]).

To explain differences in learning outcomes between simultaneous/“early” and sequential/“late” L2 learners, Hernandez, Li, and MacWhinney [54] developed the Competition and Entrenchment Model. An important element in this model is how strongly the first language (L1) is consolidated in memory and how automatized its use (also called “entrenchment”) is during L2 acquisition [55]. The model proposes that during language production there is competition between L1 and L2. That is, to select a word from L2, it needs to get more activation than its equivalent in L1. When a word within a language is activated, it spreads its activation to other related words. The more entrenched a language is, the more solidified the bonds between those words and the faster a word will reach a level of activation high enough to be selected for production. However, this also means that when there is a difference in entrenchment between the known languages (e.g., because one language has been acquired earlier) the words in the stronger language will be activated more easily than the words in the weaker language. When this happens, the speaker experiences intrusion of the stronger language into the weaker language. The model proposes that the more solidified, or entrenched, the L1 is at the moment of learning a new language, the more interference the learner will experience from the L1. This predicts that “late” bi-/multilinguals might need even more cognitive control than early bi-/multilinguals in order to solve the higher degree of interference between their languages.

Recent work has investigated whether learning a new language after early childhood, that is, during middle childhood or later, could lead to cognitive advantages [37,44,45,56,57,58,59,60,61]. The results are mixed. Luk et al. [44] found that early bilinguals outperformed late bilinguals and monolinguals on the incongruent trials of a flanker task. Late bilinguals and monolinguals performed comparably. Luk et al. [44] interpreted their results as suggesting that the bilingual advantage is a practice effect, that is, that longer experience with controlling two languages is associated with greater cognitive control abilities. Pelham and Abrams’ [46] early and late bi-/multilinguals both outperformed monolinguals on the attentional network task (ANT), but there was no difference between the two bi-/multilingual groups. On the other hand, Tao et al. [45] found faster conflict resolution in late bi-/multilinguals compared to early bi-/multilinguals on the ANT, which is consistent with the prediction of the Competition and Entrenchment Model [54]. Lastly, Bak et al. [57] found that late bilinguals differing in the age at which they had started learning the language outperformed monolinguals on an inhibition task but not on a switching task.

Interestingly, newer work on the role of age in SLA puts forward a similar perspective when it challenges the traditional view of “the earlier the better” or a “critical period” [62], as it seems that the amount and quality of input plus the opportunities to practice the language play a major role in L2 development and success, in particular, in foreign language contexts (e.g., [51,63,64]; see Oliver and Azkarai [65] for a review). Another related aspect of SLA, that might be relevant but that has not figured prominently in research into cognitive abilities, is that language learning in the four modalities (i.e., speaking, listening, writing, and reading) takes different developmental paths. Mimicking L1 development and teaching practice, L2 learning often starts with oral (listening and speaking) before written skills (reading and writing) and receptive skills (listening and reading) precede productive skills (speaking and writing) (cf. [66]). Among them, particularly, writing has received growing interest in both L1 and L2 research as it seems to play an important role in learning [67] and cognitive growth in general, which might best compared to learning a musical instrument (cf. [68]).

To the best of our knowledge, there is only one study into cognitive abilities that acknowledged these insights from SLA research: Fyndanis et al. [37] investigated sequential bi-/multilinguals who acquired their first foreign language after the age of five using bi-/multilingualism as a continuous variable depending on foreign language proficiency, usage patterns and number of known languages, and, innovatively, factoring in all four modalities (speaking, listening, writing, reading). The participants completed a non-verbal Stroop task, trail making task, and different digit span tasks. Significant correlations emerged between a proficiency-based degree of bi-/multilingualism in speaking, listening and reading on the one hand and performance on a digit ordering span task (measure of working memory) on the other hand. Similarly, a significant correlation between the number of known languages and digit span task performance was found. These results suggest that, in sequential bi-/multilinguals, foreign language proficiency might contribute more to a cognitive advantage than foreign language use, and that not only speaking but also other language modalities might contribute to a multilingualism related cognitive advantage.

### 1.2. The Current Study

The literature reviewed above, shows that research so far has provided inconclusive evidence as to whether bi-/multilingualism leads to a cognitive advantage and what factors might contribute to it. In particular, the role of different modalities for cognitive control has received little attention (Fyndanis et al. [37] being one of the few exceptions). This exploratory study does not compare monolinguals with bi-/multilinguals, but only focuses on multilinguals investigating the impact of different degrees of multilingualism on cognitive abilities. In particular, the current study aims to contribute to the ongoing debate by addressing three questions that have not been answered yet in a definitive way:

(1) Does learning one or more foreign languages after early childhood (i.e., after the age of five) enhance cognitive abilities such as executive functions and attention?
(2) If the answer to question (1) is positive, do language modalities other than speaking (i.e., listening, writing, and reading) contribute to the cognitive advantage?
(3) Is it proficiency-based or amount-of-use-based bi-/multilingualism (or both) that confer(s) greater cognitive abilities?

Following suggestions by Luk and Bialystok [48], the current study will treat bi-/multilingualism as a continuous variable. In other words, each participant will be classified as more or less bi-/multilingual based on language proficiency and usage patterns in the speaking, listening, writing, and reading modalities (i.e., the higher the proficiency and the greater the amount of use of foreign languages in each language modality, the more bi-/multilingual a speaker is).

To the best or our knowledge, this is one of the few studies that have addressed sequential multilingualism as a continuous variable [37]. Our study expands on this earlier work as we will focus on inhibition, switching, and disengagement of attention [8], while including different possible confounding variables. If bi-/multilingualism indeed influences executive functions, it is expected that the higher the degree of multilingualism, the better the inhibition, switching, and attentional disengagement abilities. However, given that some well-controlled studies found null results on “simple” executive function tasks (e.g., [8,43]), a possible outcome could also be that bi-/multilingualism only enhances attentional disengagement. Following Fyndanis et al. [37], we would tentatively expect results for proficiency-based measures to be stronger than those for amount-of-use-based measures. Moreover, based on studies showing that (1) not only speaking, but also attending to more than one language can confer a bilingual advantage in executive functioning [32,33,34], (2) during reading in L2, both languages of bilinguals are activated [69], (3) high proficiency in reading in foreign languages is related to enhanced cognitive abilities [37], and (4) modalities such as reading and writing recruit shared subskills (e.g., [70]), we would expect all four modalities to matter when it comes to dimensions of bi-/multilingualism that impact cognitive performance.

## 2. Materials and Methods

### 2.1. Participants

Native Dutch university students who had learned their first foreign language after the age of five were recruited via the database of our lab to participate in our study in exchange for a financial reward. Before the study, ethical approval was granted by the researchers’ Ethical Assessment Committee and participants signed for informed consent before they started the experiment. In total, 66 participants were tested, all with normal or corrected-to-normal vision and none of them with neurological diseases or any psychological disorders. During the experiment, twelve participants reported that they had learned their first foreign language before the age of 5 or grew up with a dialect. For this reason, they were excluded from analyses. Two participants, who were not born in the Netherlands, had moved to the Netherlands at a few months of age and were raised in Dutch. Therefore, we decided that they could remain within the final cohort of 54 participants. All participants had acquired their L2 (i.e., the language they judged themselves to be the most proficient in after their mother tongue) at a mean age of 9.73 years (SD = 1.67; range: 5–13 years). Foreign languages that were known by the participants were: Afrikaans, Arabic, Danish, English, Esperanto, French, German, Ancient Greek, Italian, Latin, Spanish, Swedish and the Dutch dialects Zeeuws and Maastrichts. All but one participant reported English as their best L2 (see Table 1 for further demographic information). Most participants were bachelor students enrolled in various programs, including artificial intelligence, history, medicine, communication, linguistics, and English language and culture. For the vast majority of participants, the curriculum was in Dutch (only for students enrolled in language bachelor programs and for a few research graduate students the curriculum was in English).

### 2.2. Tasks

#### 2.2.1. Demographic Measures

##### Raven’s Standard Progressive Matrices

To test nonverbal intelligence, participants completed a shortened version of the Raven’s Standard Progressive Matrices. To increase test efficiency and feasibility, we used the shortened 9-item version created by Bilker et al. [71] based on the original 60-item version [72] with a correlation of 0.9836 with the original test. Our participants received a paper booklet with a pattern on each page that had one missing piece and were asked to complete the pattern by choosing the correct piece from a set of 7 or 8 options by writing their answer on an answer sheet. Participants could use as much time as needed to complete the task, in which items became increasingly more difficult. Typically, the task took 15 min. Scores consisted of the number of correct answers.

##### Language and Social Background Questionnaire

To compute bi-/multilingualism related variables, we relied on self-reported data using a comprehensive language and social background questionnaire. It has been found that self-estimated language skills significantly correlate with objective measures (e.g., [73,74,75]). Ideally, objective measures should have been employed; however, given the time constraints and available resources, it was beyond the scope of this study to employ objective measures to estimate the participants’ proficiency in twelve languages (see Section 2.1) and compute amount-of-use-based degrees of multilingualism.

We merged the Language and Social Background Questionnaire developed by Anderson, Mak, Keyvani Chahi, and Bialystok [36] and a Norwegian version of this questionnaire adapted by Fyndanis, Lind, Norvik, and Simonsen [76]. Of the 21 questions, the first 12 asked about background information (e.g., age, years of education, parents’ education, and lifestyle activities that might affect executive functions, such as the weekly amount of hours spent playing musical instruments, sports, and computer games). Nine further questions targeted the linguistic background, such as the number of known languages, when and where these languages were learned, the respective proficiency and how often each language was used. Questions about proficiency and use asked for each of the four language modalities (speaking, listening, writing, reading) separately, and participants were asked to rate their proficiency for each known language and for each modality on a scale of 1 (low) to 10 (high). For language use, participants should indicate how much time they had spent on using each of their known languages in the past year by dividing 100% between their languages for each modality.

The questionnaire’s information was used to determine proficiency-based and usage-based degrees of multilingualism for each participant, keeping proficiency and use separate for each of the four modalities. In addition, we calculated different indices for productive modalities (speaking and writing), receptive modalities (listening and writing), oral modalities (speaking and listening), written modalities (writing and reading), and all modalities together (speaking, listening, writing and reading). We determined the sum of self-rated proficiency and use by adding up the scores of all known foreign languages, the mean of proficiency scores for all known foreign languages, and the proficiency and use score of the L2. For example, a hypothetical participant might know Dutch (L1), English (L2), and French (L3). Her/His self-rated proficiency and use would consist of the following scores: proficiency Dutch 10, proficiency English 8, proficiency French 4, use Dutch 60%, use English 30%, use French 10%, resulting in a sum proficiency score of L2 + L3 = 12; a mean proficiency score of (L2 + L3)/2 = 6; and the sum use score of L2 + L3 = 40%.

#### 2.2.2. Cognitive Tasks

##### Flanker Task

The flanker task (adapted from Eriksen and Eriksen [77], and Grundy and colleagues [8]) was used to measure both inhibition and disengagement of attention. Stimuli were presented using ZEP [78], a system for implementing and running psycholinguistic experiments, at the center of a 19-inch computer screen with refresh rate was 60 Hz at a distance of approximately 60 cm from the participants, subtending a horizontal visual angle of 6.9°. Stimuli remained on the screen until participants responded. Response-to-stimulus interval (RSI) was set to 250 ms, because this was the smallest RSI at which an effect was found for disengagement of attention by Grundy et al. [8]. Given this short interval, no fixation cross was needed.

During the flanker task, participants were presented with a horizontal string of arrows. They were instructed to keep their attention on the middle arrow and to indicate as quickly and as accurately as possible whether it was pointing to the left or to the right by pressing the corresponding button on a button box. The task consisted of congruent, incongruent and neutral trials. During congruent trials, all arrows pointed to the same direction, whereas in incongruent trials, the middle arrow pointed in the opposite direction from the surrounding arrows. The neutral items consisted of an arrow surrounded by horizontal lines. In order to give a correct response on an incongruent trial, participants needed to inhibit their attention to the interfering surrounding arrows.

The task started with 12 practice items on which the participants received feedback. The practice items consisted of four neutral, four congruent, and four incongruent items. After the practice round, the actual task started. The task consisted of 120 neutral and 240 test items (120 congruent and 120 incongruent) which were presented in two separate blocks. Whether the first block consisted of neutral or test items, was counterbalanced between participants. The test items were pseudorandomized: there could not be more than four consecutive items with the same type of congruency. Participants could take a break at the end of the first block and halfway of the block with test items.

Accuracy and reaction time in milliseconds on the different trial types were recorded. Because inhibitory control is necessary during the incongruent but not during the congruent trials, participants are expected to make more errors and to show a slower reaction time on the incongruent trials. Based on reaction times, the flanker effect and the sequential congruency effect (SCE) were calculated for each participant. The flanker effect is an indication of the time it takes a participant to resolve conflict caused by the surrounding arrows, and calculated by looking at the proportional increase in reaction time in incongruent trials compared to congruent trials: (incongruent–congruent)/congruent. The SCE indicates the extent to which a participant is affected by previous trial congruency. To calculate the SCE, a distinction was made between four types of trials: Incongruent trials preceded by a congruent trial (cI-trials), congruent trials preceded by a congruent trial (cC-trials), incongruent trials preceded by an incongruent trial (iI-trials) and congruent trials preceded by an incongruent trial (iC-trials). C- and i-flanker effects were computed by calculating the proportional increase between cC and cI trials ((cI–cC)/cC) and between iC and iI trials ((iI–iC)/iC), respectively. The SCE was computed by subtracting the i-flanker effect from the c-flanker effect.

##### Trail Making Task

The trail making task, used to measure switching [79], consisted of two parts. In the first part, participants received a sheet of paper with circles containing the numbers 1 to 25 arranged in random order over the sheet. They had to connect the numbers in ascending order as quickly and as accurately as possible and without lifting the pen from the paper. This part functioned as a neutral condition to assess baseline speed. In the second part of the test, participants were presented with a sheet of paper with the numbers 1 to 12 and the letters A to L arranged in random order over the sheet. Those had to be connected by alternating between letters and numbers in ascending order (e.g., 1-A-2-B etc.). Again, they had to do this as quickly and as accurately as possible without lifting the pen from the paper. This part measured switching between mental sets of letters and numbers. Any mistakes had to be corrected immediately or were pointed out by the experiment leader if the participants did not notice their mistake themselves. Correcting of mistakes added to the time of completion.

For both parts, the time it took the participants to complete them was measured in seconds. The switching cost was calculated by looking at the proportional increase in time of completion between the neutral and the switching part: (switching—neutral)/neutral.

### 2.3. Procedure

All participants performed the aforementioned tasks individually in a sound proof booth in the following order: Flanker task, trial making task, Raven’s progressive matrices task, questionnaire. The experiment took 30 to 45 min.

### 2.4. Statistical Analyses

Using SPSS Statistics, 25 correlational analyses between the different measures of degree of multilingualism and scores on the tasks were performed in order to identify relations between foreign language proficiency and use in the different modalities and combinations of modalities on the one hand and performance on the flanker and trail making test on the other hand. We used Spearman correlations because not all variables were normally distributed. Based on the correlation result, backward linear regressions were performed containing the proficiency- and use-based multilingualism variables that were significantly correlated with task performance to see if the relations between degree of multilingualism and performance on cognitive tasks would still hold when controlled for potential confounding factors and to indicate which other factors contribute to performance on cognitive tasks. Accordingly, task performance was added as dependent variable and the multilingualism related variables together with potential confounding factors (i.e., years of formal education, SES, sports (hours/week), music instruments (hours/week), video games (hours week) and Raven’s matrices score) were used as predictor variables. Assumptions of linearity, multicollinearity, homoscedasticity, and normality of the residuals were checked and fulfilled.

## 3. Results

### 3.1. Outlier Analyses and Descriptive Statistics

Prior to data analyses, incorrect responses were removed from the flanker task data. To reduce the influence of extreme values, remaining reaction times (RTs) on the flanker task were winsorized at 3 standard deviations (SD) from the participant’s mean for each condition (2.06% of the total amount of correct trials), meaning that each value 3 SD above the mean or 3 SD below the mean were replaced with a value corresponding to exactly 3 SD above/below the mean. On the trail making task, two of the 54 participants were removed from data analysis: One because the participant had dyscalculia and one because the participant had made a mistake during the task. Since correcting mistakes would influence the time it takes to finish the task, this participants’ time of completion was unreliable.

On average, participants knew 3.59 foreign languages (SD = 0.96; range: 1–5) and used 1.93 foreign languages in at least one modality (SD = 1.03; range 1–5). Table 2 shows the descriptive statistics of foreign language proficiency and use for the different measures of degree of multilingualism.

Table 3 reports on the descriptive statistics for performance on the flanker task and trail making task. Accuracy measures on the flanker task are towards ceiling and are therefore not analyzed further.

On the flanker task, mean RT on neutral trials was significantly lower than on congruent and incongruent trials (congruent: F(2, 18932) = 585.02, *p* < 0.001; incongruent: F(2, 18932) = 585.02, *p* < 0.001); mean RT on congruent trials was significantly lower than on incongruent trials (F(2, 18932) = 585.02, *p* < 0.001); incongruent trials following a congruent trial (cI-trials) took on average significantly more time than incongruent trials following another incongruent trial (iI-trials) (F(3, 12253) = 86.63, *p* < 0.001); iI-trials took longer than congruent trials following an incongruent trial (iC-trials) (F(3, 12253) = 86.63, *p* < 0.001); and the iC-trials took significantly longer than congruent trials following another congruent trials (cC-trials) (F(3, 12253) = 86.63, *p* < 0.001). Thus, RTs were slowest on cI-trials and fastest on cC trials.

On the trail making task, participants were faster during the neutral condition than during the switching condition (F(1, 102) = 116.61, *p* < 0.001).

For potential confounding variables, we checked for correlations with performance on the cognitive tasks (see Table 4), of which only weekly hours spent on gaming showed a significant negative correlation with the trail making task, meaning that the more hours a participant spent on playing computer games, the smaller his/her switching cost (r_s_ (52) = −0.36, *p* = 0.01).

### 3.2. Foreign Language Proficiency and Cognitive Performance

Results of Spearman correlations between proficiency-based measures of degree of multilingualism and performance on the different cognitive tasks are reported in Table 5.

#### 3.2.1. Flanker Task

##### Inhibition

As reported in Table 5, the correlations indicated significant or marginally significant negative associations between mean foreign language proficiency in all separate modalities and combinations of modalities and the flanker effect, indicating that the higher the mean foreign language proficiency, the lower the flanker effect and thus the smaller the cost of resolving conflict caused by interfering stimuli. Relations between the sum of foreign language proficiency and L2 proficiency in all separate modalities and combinations of modalities and the flanker effect were not significant.

The multilingualism related variables that were significantly correlated with the flanker effect were selected for backward regression analyses. Different potential confounding variables, such as years of formal education, hours per week spent on sports, music instruments and video games and performance on the Raven’s matrices test were added as predictor variables in the model. Results of the significant models explaining the most variance in performance on the flanker test are presented in Table 6.

Years of education is a significant predictor variable in all models (β ranging from −0.29 to −0.33; *p* ranging from 0.01 to 0.03). Of the multilingualism related variables, only mean writing proficiency (β = −0.26, *p* = 0.05) and combined proficiency of all modalities (β = −0.26, *p* = 0.05) were significant predictor variables of the flanker effect when controlled for confounding variables. These results indicate that the higher the writing proficiency and the proficiency in all modalities together, the lower the flanker effect and, hence, the better the performance. They also indicate that years of education is an important confounding variable.

##### Disengagement of Attention

Spearman correlations indicated significant positive relations between SCE and L2 proficiency in the listening modality and the receptive modalities (listening: r_s_(54) = 0.31, *p* = 0.03; receptive modalities: r_s_(54) = 0.29, *p* = 0.04). This indicates that the higher the L2 proficiency in these modalities, the higher the SCE and thus the higher the influence of previous trial congruency on current trial performance.

A negative marginally significant correlation was found between sum foreign language proficiency in the written modalities and SCE (r_s_(54) = −0.25, *p* = 0.07), suggesting that the higher the sum of foreign language proficiency in the written modalities, the lower the SCE and thus the lower the influence of previous trial congruency on current trial performance. Correlations with sum of foreign language proficiency in the other modalities and combinations of modalities pointed towards the same result (i.e., negative correlation), but did not reach significance.

The multilingualism related variables that were significantly correlated with SCE were selected for backward regression analyses. The potential confounding variables, such as years of formal education, hours per week spent on sports, music instruments, and video games and performance on the Raven’s matrices test were added as predictor variables in the model. Results of the significant model that explained the most variance in performance on the flanker test is presented in Table 7.

The results of the backward regression model show that only L2 listening proficiency is a significant predictor of SCE (β = 0.27, *p* = 0.05), suggesting that the higher the L2 listening proficiency, the higher the SCE and hence the worse the performance.

#### 3.2.2. Trail Making Task

Spearman correlations between switching cost and the different measures of proficiency-based degree of multilingualism showed marginally significant negative relations for mean proficiency in the reading modality, written modalities, and combination of all modalities (reading: r_s_(52) = −0.25, *p* = 0.08; written modalities: r_s_(52) = −0.24, *p* = 0.09; all modalities: r_s_(52) = −0.23, *p* = 0.10), suggesting that higher mean foreign language proficiency is associated with lower switching costs. Relations between switching cost and mean proficiency in the remaining modalities and combinations of modalities pointed in the same direction (i.e., negative correlation) but failed to reach significance.

Correlations with L2 proficiency indicated negative marginally significant relations in the reading modality and receptive modalities (reading: r_s_(52) = −0.26, *p* = 0.06; receptive modalities: r_s_(52) = −0.25, *p* = 0.08). These relations indicate that higher L2 proficiency is associated with lower switching costs in the trail making task. Correlations in the speaking, listening, written, oral, and combination of all four modalities pointed towards the same direction (i.e., negative correlations) without reaching significance.

The reported marginally significant relations should be interpreted with caution, since weekly number of hours spent on playing computer games is also negatively correlated with performance on the trail making task, such that gaming might explain part of the variation.

### 3.3. Foreign Language Use and Cognitive Performance

As reported in Table 8, none of the correlations between measures of cognition and usage-based degree of multilingualism were significant.

### 3.4. Summary of Results

To summarize, correlational analyses showed that higher mean proficiency in speaking, listening, writing, reading, productive modalities (i.e., speaking and writing combined), receptive modalities (i.e., listening and reading combined), oral modalities (i.e., speaking and listening combined), written modalities (writing and reading combined), and all modalities (i.e., speaking, listening, writing and reading combined) is related to better inhibitory control on the flanker test (i.e., a lower flanker effect). Backward linear regression models showed that, for mean writing proficiency and mean proficiency in all modalities combined, this relation still holds when controlling for confounding variables. Significant correlations were also found between SCE and L2 listening and receptive modalities proficiency, suggesting that the higher the L2 proficiency in these modalities, the worse the ability to disengage attention from previous trials (i.e., higher SCEs). This result was confirmed by a backward regression model containing only L2 listening proficiency as a predictor variable. However, models containing confounding variables were not significant. Neither correlations between performance on the trail making task and proficiency-based multilingualism variables nor correlations between usage-based variables and any cognitive measure were significant.

## 4. Discussion

This exploratory study aimed to contribute to the ongoing debate about the impact of bi-/multilingualism on cognitive abilities. In particular, we addressed three research questions: (1) Does learning one or more foreign languages after early childhood (i.e., after the age of five) enhance cognitive abilities such as executive functions and attention?; (2) If the answer to question (1) is positive, do language modalities other than speaking (i.e., listening, writing, and reading) contribute to the cognitive advantage?; (3) Is it proficiency-based or amount-of-use-based bi-/multilingualism (or both) that confer(s) greater cognitive abilities? The cognitive abilities we focused on were inhibition, switching, and disengagement of attention. To address these questions, we treated bi-/multilingualism as a continuous variable, computing different degrees of bi-/multilingualism for each participant based on foreign language proficiency and amount of use in each of the four language modalities, that is, speaking, listening, writing, and reading. Great care was taken to control for confounding factors: We only tested nonimmigrant participants (for the potential role of immigration status, see [40,41]) and investigated the impact of bi-/multilingualism while taking into account other variables that may have an effect on cognitive performance, such as aspects of lifestyle (e.g., number of hours spent on playing video games, sports, and music instruments) and demographic variables (e.g., socio-economic status, education).

Our first research question asked whether learning one or more foreign languages after early childhood enhances cognitive abilities such as executive functions and attention. The present study produced mixed results. On the one hand, we found no significant effect of bi-/multilingualism on switching, but we did find a positive effect of mean proficiency in foreign languages in the listening and writing modalities on inhibition. In other words, the higher the proficiency in foreign languages, the greater the inhibitory control. This result is consistent with studies that found similar results in “late” bi-/multilinguals (e.g., [61]), as well as with the view that not only simultaneous/early bi-/multilingualism but also sequential bi-/multilingualism leads to cognitive advantages. Importantly, it was the mean proficiency in foreign languages, not L2 proficiency, that enhanced inhibition, which suggests that knowing only one foreign language might not be enough to enhance cognitive performance. Rather, our data give support to the view that a bilingual advantage in inhibition emerges only when a certain threshold in the proficiency of each foreign language is reached. Some earlier work has shown that speaking more than two foreign languages contributes to the cognitive reserve in elders, whereas speaking two languages does not (e.g., [80,81]).

Based on our data, we cannot rule out the possibility that the relationship between proficiency-based measures of bi-/multilingualism and inhibition are bidirectional. In other words, it may be that people with enhanced components of executive functioning, such as inhibition, are more likely to become proficient in foreign languages, and that the process of learning foreign languages and achieving desirable levels of proficiency further enhances executive functioning. It has already been found that cognitive constructs such as working memory are critically involved in different aspects of foreign language learning (e.g, [82,83,84]). On the other hand, work investigating the impact of intensive foreign language learning on cognitive abilities (e.g., [56]) has shown that learning foreign languages positively impacts cognitive performance. Exploring the “immediate” impact of foreign language learning on cognitive abilities is a promising line of research, as it enables reliably establishing a baseline, making pre–post comparisons, and causal inferences (see [85]).

On the other hand, we found a negative effect of L2 listening proficiency on SCE, meaning that the higher the L2 listening proficiency, the worse the ability to disengage attention. Taken together, our results are at odds with Grundy et al. [8], who tested young adults and found a positive effect of bilingualism on disengagement of attention (SCE), but not on inhibition (flanker effect). The authors suggested that a bilingual advantage is more likely to emerge in complex measures such as SCE than in “simple” cognitive measures such as the flanker effect (for similar findings, see [86]; and for similar suggestions, see [7]). Similarly, Duñabeitia et al. [26] concluded that bilingual advantages cannot be found on simple conflict tasks.

In our study, it is hard to account for the negative effect of L2 listening proficiency on disengagement of attention. However, the combination of the positive impact of mean proficiency-based (not of L2 proficiency-based) degree of multilingualism on inhibition and of the negative impact of L2 proficiency-based degree of multilingualism on disengagement of attention suggests that only those speakers who have reached a certain proficiency threshold in more than one foreign language show a cognitive advantage, which, in our sample, emerges in inhibition only. It might be that a cognitive advantage in switching can only emerge if speakers often switch between languages within the same context (but see [24]), which was not the case with our nonimmigrant university student participants. The limited switching between languages within the same context, coupled with the fact that young adults are at their peak of cognitive performance (e.g., [9]), may have caused nonsignificant effects of bi-/multilingualism measures on switching. Another reason why no effect of bi-/multilingualism on switching was found may be that we employed a quite simple switching task (i.e., the Trail Making test).

The second research question we wanted to establish—given a positive answer to question 1—is whether language modalities other than speaking (i.e., listening, writing and reading) contribute to the cognitive advantage. To date, the bulk of studies implicitly assumed that a cognitive advantage comes from speaking at least two languages—ignoring other language modalities. Regression analyses of our data showed that, when it comes to the impact of proficiency-based degree of multilingualism on cognitive performance, both listening and writing matter. Mean proficiency in these two modalities had a marginally significant positive effect on inhibition. Importantly, this was found after having controlled for other non-bilingualism related factors that may contribute to cognitive performance (i.e., education, physical activity, playing instruments and video games, non-fluid intelligence). The result for listening is consistent with studies reporting that bilingual preverbal infants outperform monolingual infants on tasks tapping into attention or executive control (e.g., [32,33]). To the best of our knowledge, no earlier work has revealed specific effects for writing, which could be seen as the most controlled process of language, similar to knowing a musical instrument [68], and has been related to increased learning in L2 research (see [67]). Future work will need to establish whether this finding can be substantiated.

The third question addressed whether it is proficiency-based or amount-of-use-based bi-/multilingualism (or both) that confer(s) greater cognitive abilities. Results suggest that proficiency-based multilingualism contributed more to cognitive abilities such as inhibition than use-based multilingualism. In fact, we did not find any effects of amount of use-based multilingualism on cognitive performance. Luk et al. [44] viewed the bilingual advantage as a practice effect: The more one has practiced/used two languages, the greater the bilingual advantage. The lack of such an effect in our study may be due to the relatively limited variation amongst participants in the relevant variables (i.e., amount of use-based degrees of multilingualism). Most participants reported to use their L1 (Dutch) most of the time. Our results suggest that a relatively high proficiency in more than one foreign language can enhance components of executive functioning such as inhibition. This is in line with Vega-Mendoza et al. [61] and Xie and Pisano [87], who showed that higher foreign language proficiency is related to better performance on cognitive tasks. Our data are also consistent with Fyndanis et al. [37] who used a design similar to that of the present study and only found significant effects of proficiency-based degree of multilingualism on cognitive abilities such as verbal working memory and verbal short-term memory.

Lastly, there is evidence that bi-/multilingual effects on cognition often or predominantly emerge in outlying responses, and that bi-/multilingualism related effects on cognitive performance can be reduced or eliminated by applying trimming procedures (e.g., [88]). In the current study, as mentioned in Section 3.1, RTs on the flanker task were winsorized at three SDs from the participant’s mean for each condition. Following a reviewer’s suggestion, we addressed what effect the winsorization procedure had on our dataset by also performing correlational and regression analyses (similar to those reported in the Results section) on the unwinsorized flanker/SCE data. The “unwinsorized results” (presented in Appendix A Table A1, Table A2, Table A3 and Table A4) were largely aligned to the “winsorized results” (see Section 3).

### Limitations and Future Research

Although the present exploratory study addressed important research questions controlling for a number of potential confounds and treating bi-/multilingualism as a continuous variable, it also suffers some limitations. The main limitation relates to the sample size. In future research, we will strive to recruit and test much larger numbers of participants, which will ensure statistical power [89]. Another limitation is the use of the Trail Making test, which is perhaps quite easy and lacks sensitivity when it comes to testing young adult participants, who are presumably at the peak of their cognitive performance (e.g., [9]). Moreover, the Trail Making test involves inner speech; thus, it is not a purely non-verbal cognitive task. Earlier work suggested that a bi-/multilingualism related advantage in cognitive abilities is more likely to be detected on nonverbal cognitive tasks than on verbal cognitive tasks [9] because bilingualism has been found to be associated with a disadvantage in language abilities (e.g., [90]). Lastly, the present study did not collect data on patterns of switching between the languages of the participants or on the (social) contexts in which bi-/multilinguals use their languages. Such data would help more precisely describe participants’ bi-/multilingual experiences as well as investigate the role of relevant factors that were not considered here (c.f., [85,91]). In future research, we will also take these factors into account.

## 5. Conclusions

To conclude, the current study made a unique contribution to the ongoing debate regarding possible cognitive benefits of bi-/multilingualism by investigating the effects of sequential multilingualism in different linguistic modalities on cognitive performance and treating multilingualism as a continuous variable. On the one hand, the study revealed a positive effect of mean foreign language proficiency in the listening and writing domains on inhibition but, on the other hand, also found a negative effect of L2 listening proficiency on disengagement of attention. Since no effects of foreign language use on cognitive abilities were found, the results suggest that language proficiency has a bigger impact on cognition than language use. Finally, the study highlights the importance for future studies to not only look into the speaking domain but also consider other linguistic domains, such as listening, writing, and reading.

## Figures and Tables

**Table 1:** Participants’ characteristics.

	Mean (SD)	Min	Max
Male/female ratio	5/49		
Age (years)	21.07 (2.42)	18	29
Years of formal education	16.87 (1.84)	13	21
Socioeconomic status ^a^	3.96 (0.85)	2	5
Starting age L2 acquisition	9.73 (1.67)	5	13
Raven’s Matrices score ^b^	7.89 (1.00)	6	9
Sports (hours/week)	3.07 (3.03)	0	17
Computer games (hours/week)	0.40 (1.43)	0	8
Musical instruments (hours/week)	0.98 (1.93)	0	8

SD = standard deviation. ^a^ Measured by the mean level of education of both parents. Level of education was indicated on a 5-point scale. ^b^ Number of correct items out of 9.

**Table 2:** Descriptive statistics of self-reported foreign language proficiency and use for the different measures of degree of multilingualism and number of known and used languages for each modality and for all modalities together.

Proficiency		Mean (SD)	Min	Max
Speaking	Sum	17.63 (5.34)	7	32
	Mean	5.31 (1.02)	3.00	7.00
	L2	7.67 (0.91)	6	10
Listening	Sum	20.70 (5.38)	9	33
	Mean	6.26 (1.01)	4.00	9.00
	L2	8.63 (.78)	7	10
Writing	Sum	17.80 (5.27)	8	30
	Mean	5.14 (1.27)	2.60	8.00
	L2	7.98 (0.92)	6	10
Reading	Sum	23.31 (6.79)	9	40
	Mean	6.60 (1.03)	4.67	9.00
	L2	8.72 (0.68)	7	10
Productive modalities	Sum	35.43 (10.18)	15	59
	Mean	5.21 (1.08)	2.88	7.50
	L2	15.74 (1.63)	12	19
Receptive modalities	Sum	44.02 (11.48)	18	73
	Mean	6.43 (0.93)	4.50	9.00
	L2	17.35 (1.49)	14	20
Oral modalities	Sum	38.33 (10.30)	16	61
	Mean	5.79 (0.92)	3.63	8.00
	L2	16.39 (1.50)	13	20
Written modalities	Sum	41.11 (11.49)	17	70
	Mean	5.87 (1.07)	4.00	8.50
	L2	16.70 (1.60)	13	20
All modalities	Sum	79.44 (20.87)	33	131
	Mean	5.82 (0.94)	4.00	8.25
	L2	33.09 (2.92)	26	39
**Use**				
Speaking	Sum	24.63 (12.66)	10	60
	L2	18.98 (8.76)	0	40
Listening	Sum	40.46 (14.02)	10	70
	L2	33.33 (12.89)	10	70
Writing	Sum	32.22 (18.80)	0	70
	L2	28.24 (18.46)	0	70
Reading	Sum	48.98 (14.90)	10	80
	L2	42.50 (14.91)	10	80
Productive modalities	Sum	56.85 (27.55)	20	120
	L2	47.22 (24.12)	10	100
Receptive modalities	Sum	89.44 (25.60)	30	140
	L2	75.83 (25.17)	30	140
Oral modalities	Sum	65.09 (23.26)	20	120
	L2	81.20 (31.00)	20	140
Written modalities	Sum	52.31 (18.03)	20	100
	L2	70.74 (29.83)	20	140
All modalities	Sum	146.30 (49.90)	50	250
	L2	123.06 (45.17)	50	240

Sum = sum of proficiency or use scores of all known foreign languages; Mean = mean of proficiency scores of all known foreign languages; L2 = proficiency or use score of most proficient foreign language; SD = standard deviation.

**Table 3:** Descriptive statistics for the flanker task and trail making task.

		Mean (SD)	Min	Max
Flanker RT (ms) (N = 54)	Neutral	363.98 (84.74)	102	920
	Congruent	398.23 (98.70)	117	1315
	Incongruent	421.17 (98.72)	92	1099
	Incongruent preceded by congruent (cI)	424.10 (96.52)	92	1099
	Congruent preceded by congruent (cC)	386.57 (90.97)	149	1315
	Incongruent preceded by incongruent (iI)	407.51 (91.23)	184	1092
	Congruent preceded by incongruent (iC)	399.47 (96.02)	117	1315
Flanker accuracy (%)	Neutral accuracy	97.30 (2.51)	90.00	100
	Congruent accuracy	98.55 (1.44)	93.33	100
	Incongruent accuracy	96.36 (2.91)	88.33	100
Trail making (s) (n = 52)	Neutral	20.59 (4.92)	12.96	34.08
	Switching	41.64 (13.16)	21.96	85.88

SD = standard deviation.

**Table 4:** Correlations between potential confounding variables and performance on cognitive tasks.

		*Flanker Effect*	*SCE*	Trail Making *Switching Cost*
Age	r_s_ *p*	−0.06 0.68	0.06 0.68	−0.02 0.91
SES	r_s_ *p*	−0.04 0.78	0.05 0.73	−0.04 0.78
Years of formal education	r_s_ *p*	−0.11 0.44	−0.04 0.76	−0.01 0.93
Raven’s	r_s_ *p*	−0.02 0.86	0.14 0.30	−0.15 0.30
Sport (hours/week)	r_s_ *p*	0.13 0.34	0.10 0.45	0.01 0.92
Music instruments (hours/week)	r_s_ *p*	0.04 0.80	−0.16 0.24	−0.15 0.29
Gaming (hours/week)	r_s_ *p*	0.17 0.22	−0.09 0.51	−0.36 *** 0.01

*** *p* < 0.01.

**Table 5:** Spearman correlations between foreign language proficiency-based measures of degree of multilingualism and performance on cognitive tasks.

		Performance on Cognitive Tasks		
Foreign Language Proficiency-Based Measures of Degree of Multilingualism		*Flanker Effect*	*SCE*	Trail Making *Switching Cost*
**Speaking**				
Sum	r_s_ *p*	0.16 0.24	−0.14 0.32	−0.11 0.43
Mean	r_s_ *p*	−0.30 ** 0.03	0.11 0.44	−0.22 0.11
L2	r_s_ *p*	0.07 0.64	0.06 0.69	−0.10 0.48
**Listening**				
Sum	r_s_ *p*	0.20 0.15	−0.15 0.30	−0.06 0.70
Mean	r_s_ *p*	−0.34 ** 0.01	0.13 0.35	−0.14 0.33
L2	r_s_ *p*	0.04 0.75	0.31 ** 0.03	−0.23 0.10
**Writing**				
Sum	r_s_ *p*	0.07 0.63	−0.21 0.13	−0.16 0.27
Mean	r_s_ *p*	−0.28 ** 0.04	−0.02 0.89	−0.15 0.28
L2	r_s_ *p*	0.01 0.94	0.13 0.35	−0.01 0.95
**Reading**				
Sum	r_s_ *p*	0.11 0.45	−0.22 0.12	−0.15 0.30
Mean	r_s_ *p*	−0.26 * 0.06	0.05 0.73	−0.25 * 0.08
L2	r_s_ *p*	0.16 0.24	0.19 0.18	−0.26 * 0.06
**Productive modalities**				
Sum	r_s_ *p*	0.11 0.45	−0.18 0.20	−0.13 0.35
Mean	r_s_ *p*	−0.28 ** 0.04	0.02 0.88	−0.20 0.16
L2	r_s_ *p*	0.05 0.73	0.10 0.46	−0.05 0.72
**Receptive modalities**				
Sum	r_s_ *p*	0.17 0.22	−0.19 0.16	−0.12 0.41
Mean	r_s_ *p*	−0.31 ** 0.02	0.08 0.56	−0.22 0.12
L2	r_s_ *p*	0.13 0.35	0.29 ** 0.04	−0.25 * 0.08
**Oral modalities**				
Sum	r_s_ *p*	0.19 0.17	−0.15 0.29	−0.09 0.51
Mean	r_s_ *p*	−0.36 *** 0.01	0.12 0.39	−0.22 0.12
L2	r_s_ *p*	0.08 0.57	0.21 0.13	−0.17 0.24
**Written modalities**				
Sum	r_s_ *p*	0.13 0.34	−0.25 * 0.07	−0.15 0.30
Mean	r_s_ *p*	−0.28 ** 0.04	0.01 0.93	−0.24 * 0.09
L2	r_s_ *p*	0.08 0.58	0.19 0.16	−0.13 0.35
**All modalities**				
Sum	r_s_ *p*	0.15 0.29	−0.22 0.11	−0.14 0.34
Mean	r_s_ *p*	−0.33 ** 0.01	0.05 0.72	−0.23 * 0.10
L2	r_s_ *p*	0.10 0.48	0.21 0.14	−0.15 0.28

* *p* < 0.10; ** *p* < 0.05, *** *p* < 0.01; Sum = proficiency scores of all known foreign languages; Mean = mean of proficiency scores of all known foreign languages; L2 = proficiency score of most proficient foreign language.

**Table 6:** Backward regression models predicting the flanker effect from multilingualism related and potential confounding variables. Significant models explaining the most variance are presented in the table.

Variable	B	β	*p*	R^2^
Flanker effect x mean proficiency speaking			0.04	0.09
Years of education	−0.007	−0.30	0.03	
Mean proficiency speaking	−0.008	−0.18	0.17	
Flanker effect x mean proficiency listening			0.02	0.12
Music instruments (hours/week)	0.003	0.14	0.28	
Years of education	−0.011	−0.33	0.01	
Mean proficiency listening	−0.007	−0.25	0.06	
Flanker effect x mean proficiency writing			0.02	0.12
Music instruments (hours/week)	0.004	0.16	0.24	
Years of education	−0.007	−0.30	0.02	
Mean proficiency writing	−0.009	−0.26	0.05	
Flanker effect x mean proficiency productive modalities			0.03	0.11
Music instruments (hours/week)	0.003	0.14	0.28	
Years of education	−0.007	−0.30	0.03	
Mean proficiency productive modalities	−0.005	−0.23	0.08	
Flanker effect x mean proficiency receptive modalities			0.03	0.12
Music instruments (hours/week)	0.003	0.14	0.28	
Years of education	−0.007	−0.31	0.02	
Mean proficiency receptive modalities	−0.006	−0.24	0.07	
Flanker effect x mean proficiency oral modalities			0.03	0.11
Music instruments (hours/week)	0.003	0.13	0.31	
Years of education	−0.007	−0.32	0.02	
Mean proficiency oral modalities	−0.006	−0.23	0.08	
Flanker effect x mean proficiency written modalities			0.03	0.12
Music instruments (hours/week)	0.004	0.15	0.25	
Years of education	−0.007	−0.29	0.03	
Mean proficiency written modalities	−0.005	−0.25	0.06	
Flanker effect x mean proficiency all modalities			0.02	0.12
Music instruments (hours/week)	0.003	0.14	0.28	
Years of education	−0.007	−0.30	0.02	
Mean proficiency all modalities	−0.003	−0.26	0.05	

**Table 7:** Backward regression model predicting SCE from multilingualism related and potential confounding variables.

Variable	B	β	*p*	R^2^
SCE x L2 proficiency listening			0.05	0.06
L2 proficiency listening	0.02	0.27	0.05	

**Table 8:** Spearman correlations between foreign language usage-based measures of degree of multilingualism and performance on tasks measuring executive control.

		Performance on Executive Control Tasks		
Foreign Language Usage-Based Measures of Degree of Late Multilingualism		*Flanker Effect*	*SCE*	Trail Making *Switching Cost*
**Speaking**				
Sum	r_s_ *p*	0.08 0.55	−0.19 0.55	−0.02 0.88
L2	r_s_ *p*	0.17 0.23	−0.13 0.35	0.05 0.73
**Listening**				
Sum	r_s_ *p*	0.13 0.35	−0.08 0.55	−0.01 0.92
L2	r_s_ *p*	0.07 0.63	0.00 0.98	0.00 1.00
**Writing**				
Sum	r_s_ *p*	0.02 0.91	0.03 0.81	−0.05 0.75
L2	r_s_ *p*	0.08 0.57	0.08 0.55	−0.08 0.57
**Reading**				
Sum	r_s_ *p*	0.12 0.41	−0.04 0.77	−0.04 0.77
L2	r_s_ *p*	0.22 0.11	0.01 0.92	−0.04 0.76
**Productive modalities**				
Sum	r_s_ *p*	0.04 0.78	−0.06 0.67	−0.07 0.63
L2	r_s_ *p*	0.12 0.41	0.00 1.00	−0.04 0.79
**Receptive modalities**				
Sum	r_s_ *p*	0.13 0.34	−0.08 0.56	−0.06 0.67
L2	r_s_ *p*	0.19 0.18	0.01 0.92	−0.05 0.73
**Oral modalities**				
Sum	r_s_ *p*	0.13 0.36	−0.17 0.23	−0.05 0.73
L2	r_s_ *p*	0.07 0.63	−0.01 0.97	−0.07 0.65
**Written modalities**				
Sum	r_s_ *p*	0.13 0.34	−0.06 0.65	0.01 0.93
L2	r_s_ *p*	0.19 0.16	0.06 0.70	−0.12 0.41
**All modalities**				
Sum	r_s_ *p*	0.09 0.52	−0.08 0.56	−0.07 0.63
L2	r_s_ *p*	0.19 0.18	0.02 0.89	−0.08 0.57

Sum = sum of use scores of all known foreign languages; L2 = use score of most proficient foreign language.

## Appendix A

**Table A1:** Spearman correlations between foreign language proficiency-based measures of degree of multilingualism and performance on cognitive tasks (*unwinsorized Flanker data*).

		Performance on Cognitive Tasks		
Foreign Language Proficiency-Based Measures of Degree of Late Multilingualism		*Flanker Effect*	*SCE*	Trail Making *Switching Cost*
**Speaking**				
Sum	r_s_ *p*	0.17 0.23	−0.13 0.35	−0.11 0.43
Mean	r_s_ *P*	−0.29 ** 0.03	0.07 0.61	−0.22 0.11
L2	r_s_ *p*	0.08 0.59	0.10 0.47	−0.10 0.48
**Listening**				
Sum	r_s_ *p*	0.20 0.15	−0.13 0.37	−0.06 0.70
Mean	r_s_ *p*	−0.31 ** 0.02	0.13 0.34	−0.14 0.33
L2	r_s_ *p*	0.04 0.79	0.33 ** 0.02	−0.23 0.10
**Writing**				
Sum	r_s_ *p*	0.05 0.70	−0.22 0.12	−0.16 0.27
Mean	r_s_ *p*	−0.29 ** 0.03	−0.06 0.67	−0.15 0.28
L2	r_s_ *p*	0.03 0.85	0.19 0.17	−0.01 0.95
**Reading**				
Sum	r_s_ *p*	0.15 0.29	−0.23 0.10	−0.15 0.30
Mean	r_s_ *p*	−0.26 * 0.06	0.01 0.96	−0.25 * 0.08
L2	r_s_ *p*	0.16 0.25	0.20 0.16	−0.26 * 0.06
**Productive modalities**				
Sum	r_s_ *p*	0.10 0.47	−0.18 0.20	−0.13 0.35
Mean	r_s_ *p*	−0.29 ** 0.04	−0.01 0.93	−0.20 0.16
L2	r_s_ *p*	0.06 0.65	0.16 0.25	−0.05 0.72
**Receptive modalities**				
Sum	r_s_ *p*	0.17 0.23	−0.19 0.18	−0.12 0.41
Mean	r_s_ *p*	−0.30 ** 0.03	0.05 0.73	−0.22 0.12
L2	r_s_ *p*	0.13 0.37	0.30 ** 0.03	−0.25 * 0.08
**Oral modalities**				
Sum	r_s_ *p*	0.19 0.17	−0.13 0.34	−0.09 0.51
Mean	r_s_ *p*	−0.33 ** 0.01	0.09 0.51	−0.22 0.12
L2	r_s_ *p*	0.08 0.56	0.25 * 0.07	−0.17 0.24
**Written modalities**				
Sum	r_s_ *p*	0.12 0.39	−0.26 * 0.06	−0.15 0.30
Mean	r_s_ *p*	−0.29 ** 0.04	−0.03 0.86	−0.24 * 0.09
L2	r_s_ *p*	0.08 0.56	0.23 0.10	−0.13 0.35
**All modalities**				
Sum	r_s_ *p*	0.14 0.31	−0.22 0.11	−0.14 0.34
Mean	r_s_ *p*	−0.33 ** 0.01	0.01 0.92	−0.23 * 0.10
L2	r_s_ *p*	0.10 0.45	0.24 * 0.08	−0.15 0.28

* *p* < 0.10; ** *p* < 0.05; Sum = proficiency scores of all known foreign languages; Mean = mean of proficiency scores of all known foreign languages; L2 = proficiency score of most proficient foreign language.

**Table A2:** Backward regression models predicting the flanker effect from multilingualism related and potential confounding variables. Significant models explaining the most variance are presented in the table. All models are based on unwinsorized Flanker data.

Variable	B	β	*p*	R^2^
Flanker effect x mean proficiency speaking			0.05	0.09
Music instruments (hours/week)	0.003	0.13	0.31	
Years of education	−0.008	−0.32	0.02	
Mean proficiency speaking	−0.008	−0.16	0.22	
Flanker effect x mean proficiency listening			0.02	0.12
Music instruments (hours/week)	0.004	0.15	0.27	
Years of education	−0.008	−0.35	0.01	
Mean proficiency listening	−0.011	−0.23	0.08	
Flanker effect x mean proficiency writing			0.01	0.15
Music instruments (hours/week)	0.004	0.16	0.22	
Years of education	−0.008	−0.32	0.02	
Mean proficiency writing	−0.010	−0.27	0.04	
Flanker effect x mean proficiency productive modalities			0.02	0.13
Music instruments (hours/week)	0.004	0.15	0.26	
Years of education	−0.008	−0.32	0.02	
Mean proficiency productive modalities	−0.005	−0.24	0.07	
Flanker effect x mean proficiency receptive modalities			0.02	0.12
Music instruments (hours/week)	0.004	0.15	0.27	
Years of education	−0.008	−0.33	0.02	
Mean proficiency receptive modalities	−0.006	−0.23	0.08	
Flanker effect x mean proficiency oral modalities			0.03	0.12
Music instruments (hours/week)	0.003	0.14	0.29	
Years of education	−0.008	−0.33	0.01	
Mean proficiency oral modalities	−0.006	−0.22	0.10	
Flanker effect x mean proficiency written modalities			0.02	0.14
Music instruments (hours/week)	0.004	0.15	0.24	
Years of education	−0.007	−0.31	0.02	
Mean proficiency written modalities	−0.006	−0.26	0.05	
Flanker effect x mean proficiency all modalities			0.02	0.14
Music instruments (hours/week)	0.004	0.15	0.26	
Years of education	−0.008	−0.32	0.02	
Mean proficiency all modalities	−0.003	−0.25	0.05	

**Table A3:** Backward regression models predicting SCE from multilingualism related and potential confounding variables. All models are based on unwinsorized Flanker data.

Variable	B	β	*p*	R^2^
SCE x L2 proficiency listening			0.04	0.10
Sports (hours/week)	0.004	0.19	0.16	
Music instruments (hours/week)	−0.005	−0.17	0.22	
L2 proficiency listening	0.032	0.39	0.01	
SCE x L2 proficiency receptive modalities			0.05	0.06
L2 proficiency receptive modalities	0.012	0.27	0.05	

**Table A4:** Spearman correlations between foreign language usage-based measures of degree of multilingualism and performance on cognitive tasks (*unwinsorized Flanker data*).

		Performance on Executive Control Tasks		
Foreign Language Usage-Based Measures of Degree of Late Multilingualism		Flanker *Flanker Effect*	Flanker *SCE*	Trail Making *Switching Cost*
**Speaking**				
Sum	r_s_ *p*	0.10 0.49	−0.16 0.26	−0.02 0.88
L2	r_s_ *p*	0.18 0.20	−0.08 0.57	0.05 0.73
**Listening**				
Sum	r_s_ *p*	0.13 0.37	−0.09 0.54	−0.01 0.92
L2	r_s_ *p*	0.06 0.67	0.02 0.91	0.00 1.00
**Writing**				
Sum	r_s_ *p*	0.03 0.82	0.09 0.53	−0.05 0.75
L2	r_s_ *p*	0.10 0.50	0.15 0.29	−0.08 0.57
**Reading**				
Sum	r_s_ *p*	0.13 0.35	0.01 0.95	−0.04 0.77
**L2**	r_s_ *p*	0.23 0.09 *	0.08 0.55	−0.04 0.76
**Productive modalities**				
Sum	r_s_ *p*	0.06 0.69	−0.01 0.94	−0.07 0.63
L2	r_s_ *p*	0.13 0.35	0.07 0.63	−0.04 0.79
**Receptive modalities**				
Sum	r_s_ *p*	0.14 0.32	−0.05 0.70	−0.06 0.67
L2	r_s_ *p*	0.19 0.17	0.06 0.66	−0.05 0.73
**Oral modalities**				
Sum	r_s_ *p*	0.13 0.34	−0.15 0.29	−0.05 0.73
L2	r_s_ *p*	0.08 0.55	0.05 0.70	−0.07 0.65
**Written modalities**				
Sum	r_s_ *p*	0.13 0.33	−0.03 0.84	0.01 0.93
L2	r_s_ *p*	0.21 0.13	0.13 0.34	−0.12 0.41
**All modalities**				
Sum	r_s_ *p*	0.10 0.46	−0.04 0.80	−0.07 0.63
L2	r_s_ *p*	0.20 0.16	0.08 0.55	−0.08 0.57

* *p* < 0.10; Sum = sum of use scores of all known foreign languages; L2 = use score of most proficient foreign language.
